# Heat Shock Protein Beta-1 Modifies Anterior to Posterior Purkinje Cell Vulnerability in a Mouse Model of Niemann-Pick Type C Disease

**DOI:** 10.1371/journal.pgen.1006042

**Published:** 2016-05-06

**Authors:** Chan Chung, Matthew J. Elrick, James M. Dell’Orco, Zhaohui S. Qin, Shanker Kalyana-Sundaram, Arul M. Chinnaiyan, Vikram G. Shakkottai, Andrew P. Lieberman

**Affiliations:** 1 Department of Pathology, University of Michigan Medical School, Ann Arbor, Michigan, United States of America; 2 Department of Neurology, University of Michigan Medical School, Ann Arbor, Michigan, United States of America; 3 Department of Biostatistics and Bioinformatics, Emory University, Atlanta, Georgia, United States of America; 4 Michigan Center for Translational Pathology, University of Michigan Medical School, Ann Arbor, Michigan, United States of America; 5 Howard Hughes Medical Institute, University of Michigan Medical School, Ann Arbor, Michigan, United States of America; University of Minnesota, UNITED STATES

## Abstract

Selective neuronal vulnerability is characteristic of most degenerative disorders of the CNS, yet mechanisms underlying this phenomenon remain poorly characterized. Many forms of cerebellar degeneration exhibit an anterior-to-posterior gradient of Purkinje cell loss including Niemann-Pick type C1 (NPC) disease, a lysosomal storage disorder characterized by progressive neurological deficits that often begin in childhood. Here, we sought to identify candidate genes underlying vulnerability of Purkinje cells in anterior cerebellar lobules using data freely available in the Allen Brain Atlas. This approach led to the identification of 16 candidate neuroprotective or susceptibility genes. We demonstrate that one candidate gene, heat shock protein beta-1 (*HSPB1*), promoted neuronal survival in cellular models of NPC disease through a mechanism that involved inhibition of apoptosis. Additionally, we show that over-expression of wild type *HSPB1* or a phosphomimetic mutant in NPC mice slowed the progression of motor impairment and diminished cerebellar Purkinje cell loss. We confirmed the modulatory effect of Hspb1 on Purkinje cell degeneration *in vivo*, as knockdown by *Hspb1* shRNA significantly enhanced neuron loss. These results suggest that strategies to promote HSPB1 activity may slow the rate of cerebellar degeneration in NPC disease and highlight the use of bioinformatics tools to uncover pathways leading to neuronal protection in neurodegenerative disorders.

## Author Summary

Niemann-Pick type C1 (NPC) disease is an autosomal recessive lipid storage disorder for which there is no effective treatment. Patients develop a clinically heterogeneous phenotype that typically includes childhood onset neurodegeneration and early death. Mice with loss of function mutations in the *Npc1* gene model many aspects of the human disease, including cerebellar degeneration that results in marked ataxia. Cerebellar Purkinje cells in mutant mice exhibit striking selective vulnerability, with neuron loss in anterior lobules and preservation in posterior lobules. As this anterior to posterior gradient is reproduced following cell autonomous deletion of *Npc1* and is also observed in other forms of cerebellar degeneration, we hypothesized that it is mediated by differential gene expression. To test this notion, we probed the Allen Brain Atlas to identify 16 candidate neuroprotective or susceptibility genes. We confirmed that one of these genes, encoding the small heat shock protein Hspb1, promotes survival in cell culture models of NPC disease. Moreover, we found that modulating Hspb1 expression in NPC mice promoted (following over-expression) or diminished (following knock-down) Purkinje cell survival, confirming its neuroprotective activity. We suggest that this approach may be similarly used in other diseases to uncover pathways that modify selective neuronal vulnerability.

## Introduction

Selective vulnerability of specific neuronal populations is a well characterized, though often perplexing feature of many neurodegenerative diseases [1]. Most commonly, these disorders are initiated by a uniform stress to the entire CNS, such as a genetic mutation, toxic insult, or aging. However, only a subset of neurons respond to these stressors by degenerating, while others remain resistant and apparently maintain their normal function [2]. Although this phenomenon is widely observed, the underlying mechanisms remain poorly understood. Notably, the factors regulating neuronal vulnerability represent attractive therapeutic targets, with the potential to convert susceptible neuronal populations into ones that are disease resistant.

One particularly striking example of selective vulnerability is the degeneration of cerebellar Purkinje cells [3]. Purkinje cells represent the sole output of the cerebellar cortex. Loss of Purkinje cells, therefore, leads to significant deficits of motor coordination, including ataxia and tremors. Despite the apparent similarity of Purkinje cells in their morphology, connectivity, and electrophysiological properties, many cerebellar disorders affect Purkinje cells in a non-uniform way, leading to a distinct spatiotemporal pattern of loss that is reproducible not only between cases of a single disease, but across many otherwise unrelated diseases and injuries. One common pattern reveals a strong resistance of Purkinje cells in lobule X to degeneration, contrasted with the exquisite sensitivity of the anterior zone (lobules II-V), and moderate susceptibility of the intermediate (lobules VI-VII) and posterior zones (lobule VIII and rostral aspect of lobule IX). Superimposed onto this anterior-to-posterior gradient is often a pattern of parasagittal stripes in which differential vulnerability is also observed [3]. Diseases displaying the classic anterior-to-posterior gradient may arise from genetic mutations, including spinocerebellar ataxias type 1 [4] and 6 [5], late infantile neuronal ceroid lipofuscinosis [6], saposin C deficiency, a rare cause of Gaucher Disease [7], ataxia telangiectasia [8], and Niemann-Pick disease types A/B [9] and C [10]; sporadic disorders, including multiple system atrophy [11] and chronic epilepsy [12]; toxins, including alcohol [13], cytosine arabinoside [14], methotrexate [15]; hypoxia/ischemia [16, 17]; paraneoplastic syndromes [18]; and even normal aging [19]. This pattern is also seen in many spontaneous mouse mutants, including *pcd* [20], *leaner* [21], *toppler* [22], *robotic* [23], *shaker* [24], and *lurcher* [25]; or targeted mutants, such as *saposin D* knockout [26], prion protein knockout [27], and over-expression of the prion protein related gene *doppel* [28]. The fact that such a diverse array of insults leads to the same pattern of Purkinje cell death suggests that selective vulnerability of Purkinje cell subpopulations arises not from the initiating event of the disease process, but instead from differential regulation of cellular survival or death pathways in response to these injuries. We hypothesize that the identification of pathways responsible for this phenomenon will yield therapeutic targets broadly applicable to this large class of cerebellar disorders.

As a model for patterned Purkinje cell loss, we have studied murine Niemann-Pick type C1 disease (NPC). NPC is caused by mutations in the genes encoding NPC1 or NPC2 proteins, which are thought to act cooperatively in the efflux of cholesterol from late endosomes (LE) and lysosomes (LY) [29–31]. The consequence of these mutations is the accumulation of cholesterol and glycosphingolipids in the LE/LY compartment, leading to neurodegeneration by mechanisms that are not yet understood [32]. We previously demonstrated that conditional deletion of *Npc1* in Purkinje cells leads to cell autonomous degeneration that recapitulates the spatiotemporal pattern of cell loss observed in mice with germline *Npc1* deletion [33]. Further, because Purkinje cell death does not cause early mortality in these mice, we were able to follow Purkinje cell survival beyond the typical lifespan of NPC mice. During this period, the population of surviving Purkinje cells in lobule X remained stable, while neurodegeneration continued to progress in lobules II-IX, thus highlighting the strong resistance of these cells to degeneration. Given the cell autonomous nature of Purkinje cell loss in NPC, we hypothesized that this selective vulnerability arises from intrinsic biological differences that are driven by differential gene expression. To test this notion, here we used a bioinformatics approach to identify genes that are differentially expressed between disease-resistant and vulnerable Purkinje cell populations. To test the biological function of these differentially expressed genes, we used *in vitro* and *in vivo* model of NPC and characterized the ability of one of these candidate genes to protect neurons from degeneration.

## Results

### Identification of candidate genes underlying selective vulnerability of Purkinje cells

**Fig 1:**
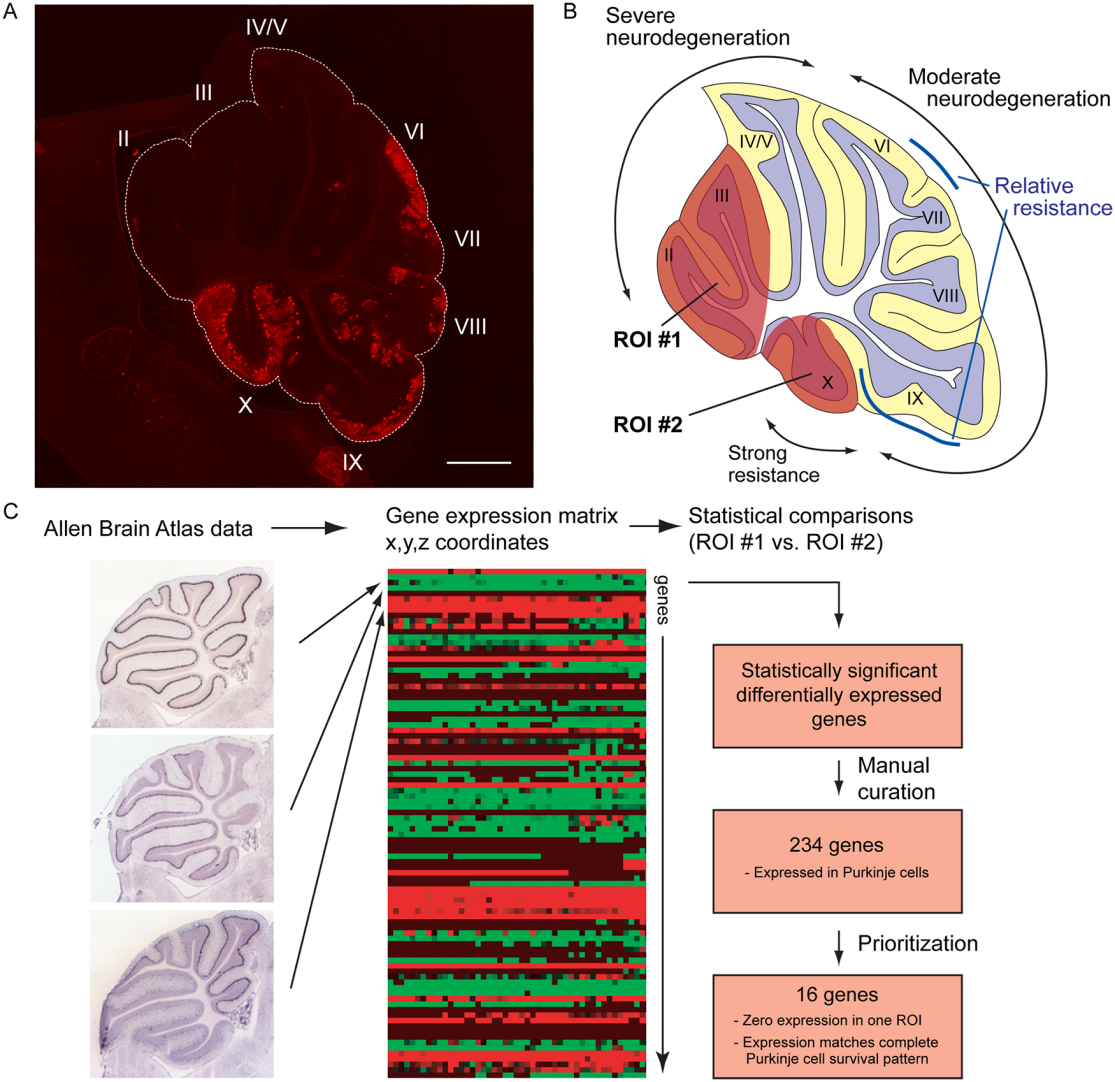
Schematic of gene expression analysis. **(A)** Calbindin staining of cerebellum from 20-week old *Npc1 flox/-*, *Pcp2-Cre* mouse, demonstrating survival pattern of Purkinje cells in midline. Scale bar = 500 μm. **(B)** Schematic of differential vulnerability of Purkinje cell subpopulations. Regions of interest (ROI) were selected to include the population that experiences the most rapid neurodegeneration (lobules II and III, ROI #1) or the population that does not degenerate (lobule X, ROI #2). **(C)** Approach to gene expression analysis. Allen Brain Atlas data was downloaded and consolidated into a single gene expression matrix. Each row represents gene expression data from a single series of *in situ* hybridization data, while each column represents a single voxel within the mouse brain. The data set was then narrowed to include only voxels lying within the defined regions of interest. To identify genes differentially expressed between regions of interest, expression data was treated analogously to replicate microarrays and subjected to standard statistical tests (*t*-test and Significance Analysis for Microarrays). The top 1000 most significant genes were accepted for manual curation to verify expression in Purkinje cells. The gene list was further narrowed to include only genes with absolute expression differences between regions of interest, and expression matching the pattern of Purkinje cell survival or death throughout the entire cerebellum.

Using mice containing a conditional null allele of the *Npc1* gene, we found that gene deletion in Purkinje cells recapitulates the spatiotemporal pattern of neuron loss observed in mice with global germline deletion of *Npc1* (Fig 1A) [10, 33]. The population of surviving Purkinje cells is located within posterior lobules of the cerebellar midline, while age-dependent progressive Purkinje cell loss is observed in anterior lobules (Fig 1B) [33]. We hypothesized that differential gene expression underlies this selective neuronal vulnerability. To search for genes differentially expressed between Purkinje cell subpopulations, we utilized the Allen Brain Atlas (Fig 1C). This resource contains quantitative three-dimensional expression data derived from *in situ* hybridizations for greater than 20,000 genes in the adult C57BL6/J mouse brain [34]. The complete gene expression dataset was downloaded and used to construct a single expression matrix with spatial coordinates and gene identifiers arrayed on separate axes. This strategy allowed us to treat the data for each location in the brain analogously to a single microarray experiment. The coordinates corresponding to cerebellar lobule X, the location of the most resistant Purkinje cells, and lobules II and III, the most highly vulnerable, were defined as regions of interest (Fig 1B). For analysis, all coordinates falling within one region of interest were treated as replicate microarray experiments. We then used bioinformatics tools developed for microarray analysis to query the Allen Brain Atlas dataset. Differential gene expression between lobules was determined by *t*-test and Significance Analysis of Microarrays (SAM) [35], followed by manual curation of *in situ* hybridization images. Manual curation was required to remove false positives created by expression in non-Purkinje cell types and technical artifacts in the archived images.

**Fig 2:**
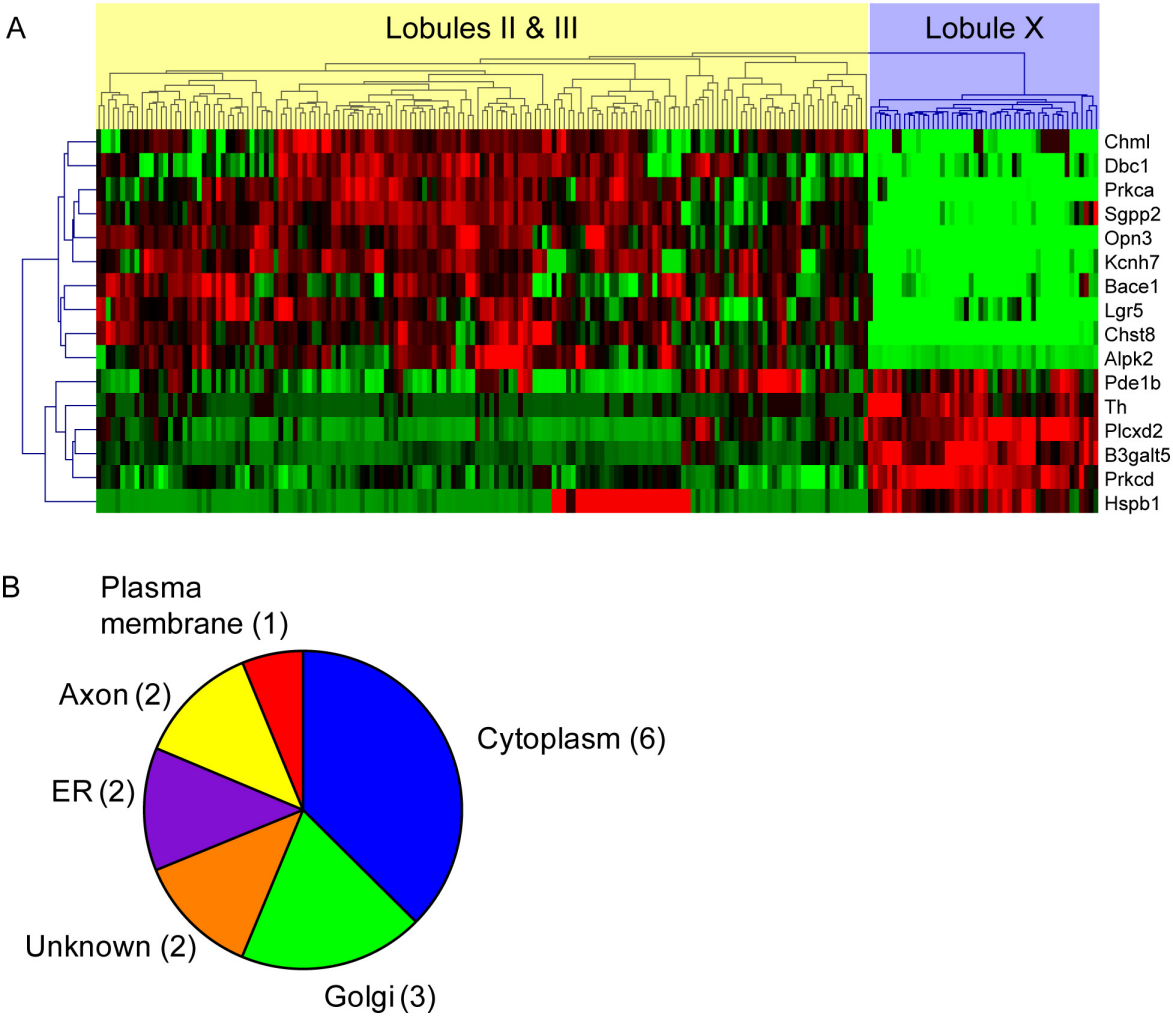
Candidate neuroprotective or pro-degenerative genes. **(A)** Hierarchical clustering of candidate genes, demonstrating strong differential expression between regions of interest. Rows, genes; columns, individual voxels within the regions of interest. Red designates higher and green designates lower expression. **(B)** Subcellular localization of candidate genes, based on GO terms and review of supporting literature.

Initial analysis revealed 234 differentially expressed genes, of which 185 were more highly expressed in lobules II and III and 49 were more highly expressed in lobule X. We next sought to prioritize this list to identify testable candidates with putative roles in promoting or preventing neurodegeneration. The Allen Brain Atlas data, being derived from *in situ* hybridizations, presented a challenge in this regard, as expression levels were regarded as semi-quantitative. Further, because expression data within each *z* plane came from the same hybridization experiment, they were not considered statistically independent samples. For these reasons, we were unable to rank the gene list by either the magnitude of differential expression or the degree of significance. Instead, we prioritized genes whose expression differences were most robust and tightly correlated with Purkinje cell survival in midline cerebellar sections. To accomplish this, we only included genes whose expression was undetectable in one region of interest, and whose expression matched or was the inverse of the survival pattern in 20 week old *Npc1 flox/-;Pcp2-Cre* mice: strong in lobule X, patchy throughout the intermediate and posterior zones, with additional sparing in the caudal aspect of lobule IX and a region spanning the caudal aspect of lobule VI and rostral lobule VII (Fig 1A). This yielded sixteen candidate neuroprotective or susceptibility genes (Fig 2A, Table 1); *in situ* hybridization images from the Allen Brain Atlas for the candidate genes highly expressed in regions of cell survival are shown in S1 Fig.

**Table 1:** Genes differentially expressed in Purkinje cells in anterior or posterior lobules.

Gene symbol	Gene name	Gene ID	Region of expression
B3galt5	UDP-Gal:betaGlcNAc beta 1,3-galactosyltransferase, polypeptide 5	93961	posterior
Hspb1	heat shock protein beta-1	15507	posterior
Pde1b	phosphodiesterase 1B, Ca2+-calmodulin dependent	18574	posterior
Plcxd2	phosphatidylinositol-specific phospholipase C, X domain containing 2	433022	posterior
Prkcd	protein kinase C, delta	18753	posterior
Th	tyrosine hydroxylase	21823	posterior
Alpk2	alpha-kinase 2	225638	anterior
Bace1	beta-site APP cleaving enzyme 1	23821	anterior
Chml	choroideremia-like	12663	anterior
Chst8	carbohydrate (N-acetylgalactosamine 4–0) sulfotransferase 8	68947	anterior
Dbc1	deleted in bladder cancer 1	56710	anterior
Kcnh7	potassium voltage-gated channel, subfamily H (eag-related), member 7	170738	anterior
Lgr5	leucine rich repeat containing G protein coupled receptor 5	14160	anterior
Opn3	opsin (encephalopsin)	13603	anterior
Prkca	protein kinase C, alpha	18750	anterior
Sgpp2	sphingosine-1-phosphate phosphatase 2	433323	anterior

We analyzed the functions of these candidate genes and their human orthologs by querying their gene ontology (GO) annotations using AmiGO [36]. The GO Term Enrichment tool revealed significant over-representation (*p*<0.01) for GO terms containing *Prkca*, *Prkcd*, and *Plcxd2*, members of the phospholipase C—protein kinase C signal transduction cascade, suggesting that this pathway is differentially regulated between regions of interest. AmiGO was also used to query the complete list of GO Biological Process terms associated with candidate genes. In support of our hypothesis that the differentially expressed genes would include regulators of cellular survival and death decisions, 5 genes were associated with cell death related annotations, including “cell death” (GO:0008219, *Dbc1* and *Hspb1*), “apoptosis” (GO:0006915, *Pde1b* and *Prkcd*), “negative regulation of apoptosis” (GO:0043066, *Hspb1*), and “induction of apoptosis by intracellular signals” (GO:0008629, *Prkca*). Furthermore, the gene product of *Sgpp2*, sphingosine 1-phosphate phosphatase 2, is likely involved in the regulation of apoptosis as well due to its hydrolysis of sphingosine 1-phosphate [37], a lipid second messenger that is a negative regulator of apoptosis [38]. Finally, we performed an analysis of cellular component annotations to determine the subcellular localization of the protein products of candidate genes (Fig 2B). The vast majority of gene products are localized outside of the endosome-lysosome system, further suggesting that selective vulnerability of Purkinje cell populations arises not from the primary site of pathogenesis in NPC disease, but from responses to cellular stress that take place elsewhere.

### *HSPB1* promotes survival of *in vitro* models of NPC disease

We next sought to directly test the extent to which candidate genes influence cell survival in models of NPC disease. For this initial analysis, we chose to study one gene that was over-expressed by lobule X Purkinje cells, heat shock protein beta 1 (*Hspb1*). This gene has been linked previously to neurodegeneration, as mutations in human *HSPB1* cause some cases of Charcot-Marie-Tooth disease and distal hereditary motor neuropathy [39]. Additionally, HSPB1 regulates multiple events that influence neuronal viability, including stability of the actin cytoskeleton, protein folding, reactive oxygen species (ROS), and apoptosis [40], and its robust expression has been documented in surviving Purkinje cells from *Npc1-/-* mice [10].

**Fig 3:**
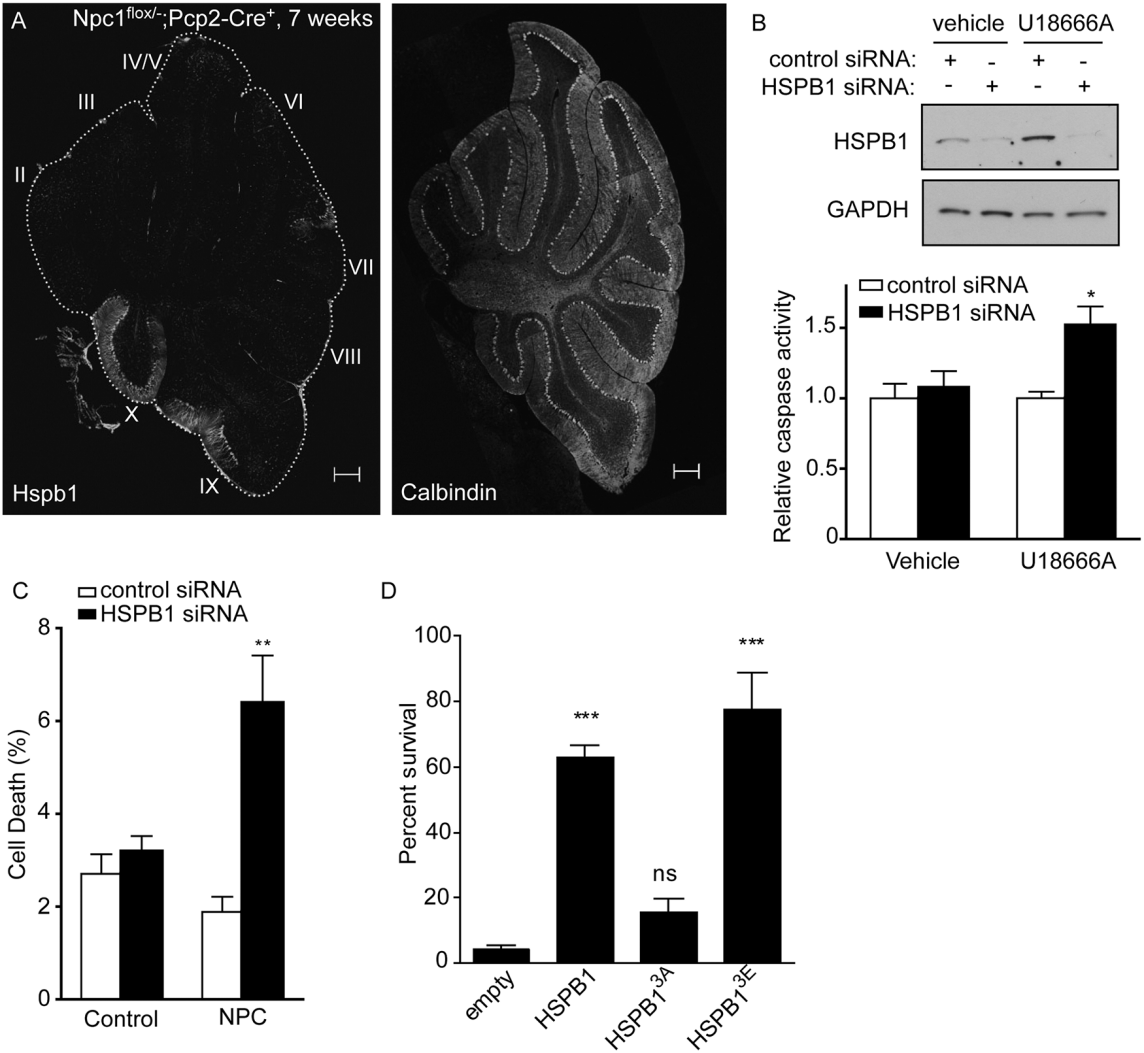
HSPB1 promotes survival in cellular models of NPC1 disease. **(A)** Expression of Hspb1 *(left panel)* and calbindin *(right panel*, Purkinje cells) in cerebellar midline of *Npc1 flox/-*, *Pcp2-Cre* mice 7 weeks. Scale bar = 200 μm. **(B)** (*Upper panel*) HeLa cells were transfected with non-targeted (NT, lanes 1 and 3) or *HSPB1* siRNA (lanes 2 and 4), then treated with vehicle (lanes 1–2) or 1 mg/ml U18666A (lanes 3–4) for 24 hr. HSPB1 expression was determined by western blot. GAPDH controls for loading. (*Lower panel*) Caspase-3 in HeLa cell lysates. Data are mean ± SEM. *p<0.05. **(C)** NPC1 patient fibroblasts were transfected with non-targeted or *HSPB1* siRNA. Cells were stained with Hoechst, and the percentage of cells with condensed chromatin was scored. Data are mean ± SEM. **p<0.01. **(D)** Primary mouse cortical neurons were transduced with wild type *HSPB1*, *HSPB1*^*3A*^, *HSPB1*^*3E*^, or empty vector, and then treated with 2.5 μg/ml U18666. XTT assay was performed 72 hrs post U18666A. Neuron survival is reported relative to vehicle treated cells. Data are mean ± SEM. ***p<0.001.

We initially sought to confirm that Hspb1 expression in mutant mice with active disease matched the pattern predicted by the Allen Brain Atlas. Strong expression of *Hspb1* was detected in lobule X Purkinje cells of *Npc1 flox/-;Pcp2-Cre* mice at 7 weeks of age, prior to the significant Purkinje cell degeneration (Fig 3A). In contrast, Hspb1 was undetectable in the more susceptible Purkinje cells of lobules II and III. To determine whether HSPB1 functions as an inhibitor of cell death pathways in NPC cell models, we knocked down its expression using siRNA. We initially treated HeLa cells with U18666A, a small molecule which induces lipid trafficking defects similar to those seen in NPC disease by binding to NPC1 and inhibiting cholesterol export [41, 42]. Knockdown of *HSPB1* in U18666A-treated cells, but not in vehicle controls, led to a significant increase of caspase activity (Fig 3B). Likewise, *HSPB1* knockdown in NPC patient fibroblasts significantly increased the percentage of cells with chromatin condensation, while *HSPB1* knockdown had no effect on control fibroblasts (Fig 3C). These results are consistent with a model in which HSPB1 prevents the induction of cell death in response to the intracellular lipid trafficking defects caused by NPC1 deficiency.

To initially test the role of HSPB1 in the survival of neurons, the cell type critical for NPC disease neuropathology [33, 43, 44], we utilized a neuronal culture model. Primary cortical neurons treated with U18666A develop filipin-positive lipid inclusions and progressive degeneration, and have been used previously to model NPC disease [45, 46]. Neurons treated with U18666A demonstrated progressive degeneration, and exogenous over-expression of *HSPB1* almost completely prevented this death (Fig 3D). To probe the mechanism of this effect, we took advantage of the fact that serine phosphorylation is critical for HSPB1-mediated protection against neuronal damage *in vitro* and *in vivo* [47]. Mutation of these residues to alanine (non-phosphorylatable) or aspartate/glutamate (phosphomimetic) has been widely used to study phosphorylation state-dependent properties of HSPB1 [40]. Transduction of U18666A-treated neurons with the phosphomimetic *HSPB1*-3E recapitulated the neuroprotective effects of wild-type *HSPB1*, while non-phosphorylatable *HSPB1*-3A was inactive (Fig 3D). We conclude that the neuroprotective effects of HSPB1 in NPC cell models are mediated by the phosphorylated species.

### *HSPB1* over-expression diminishes motor impairment and Purkinje cell loss

**Fig 4:**
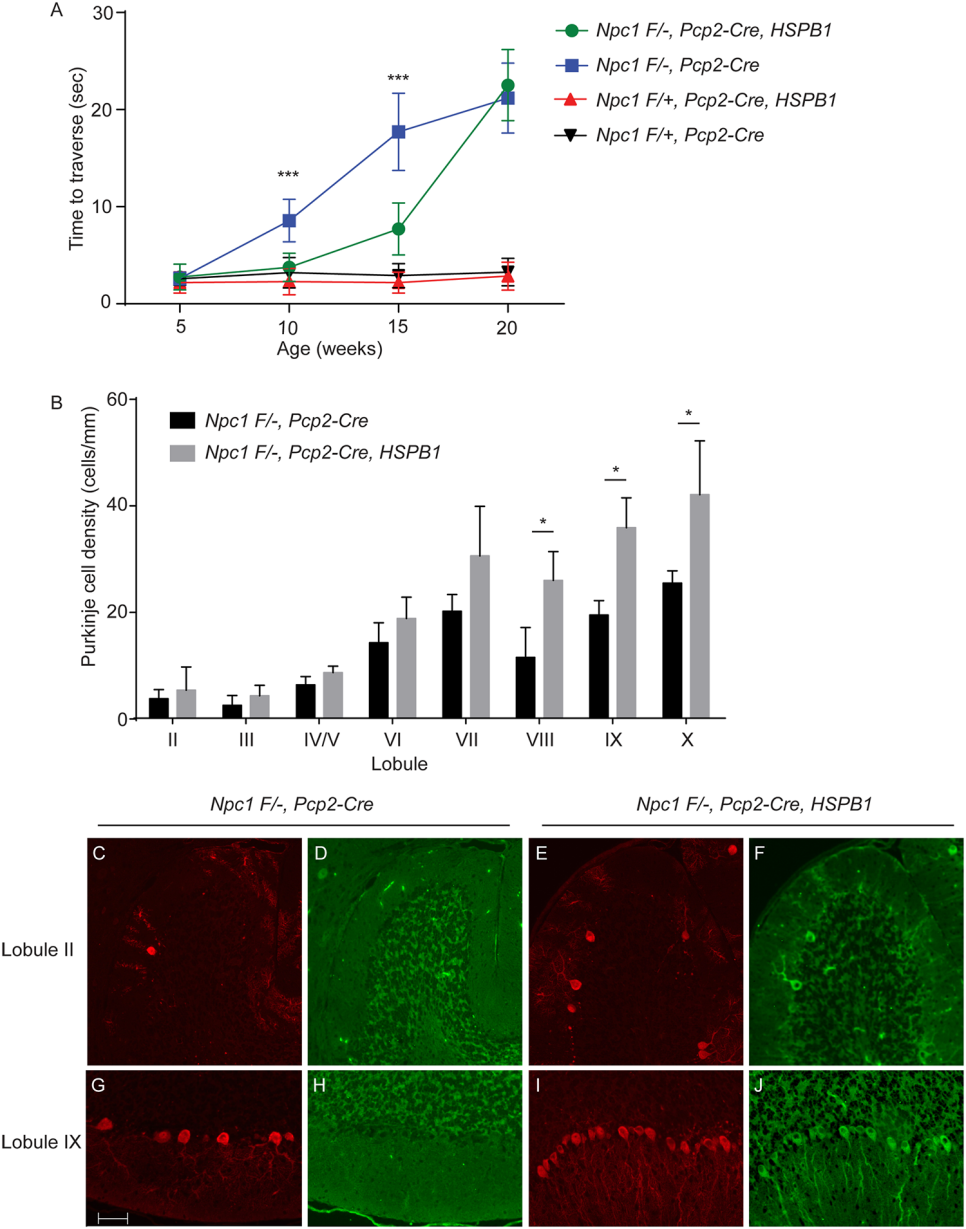
HSPB1 over-expression rescues motor impairment and Purkinje cell loss. **(A)** Age-dependent performance on balance beam indicates that transgenic *HSPB1* over-expression delays motor impairment in *Npc1 flox/-*, *Pcp2-Cre* mice. Data are mean ± SD, *n* ≥ 7 mice/genotype. ***p<0.001. **(B)** Purkinje cell density in indicated lobules of the cerebellar midline of 11-week-old mice. Data are mean ± SD, *n* = 3 mice/genotype. *p<0.05. **(C–J)** Purkinje cells (calbindin, *in red*) and transgenic HSPB1 (HA, *in green*) in anterior and posterior lobules of the cerebellar midline of 11-week-old mice. *Top row*, lobule II; *bottom row*, lobule IX. Scale bar = 50 μm.

We next sought to determine whether HSPB1 over-expression impacts Purkinje cell survival and motor impairment in NPC mice. To accomplish this, we generated mice deficient in *Npc1* only in Purkinje cells by using a previously characterized conditional null allele [33]. Cre recombinase expression driven by the *Pcp2* promoter initiated around postnatal day 6 and was present in all Purkinje cells by postnatal days 14–21 [48]. Therefore, this strategy enabled post-developmental as well as cell-type restricted deletion of *Npc1*. Expression of the hemagglutinin (HA)-tagged human *HSPB1* cDNA transgene was driven by the chicken β-actin promoter and cytomegalovirus enhancer. These transgenic mice express exogenous HSPB1 in brain, spinal cord, heart, muscle, liver, kidney, lung, and pancreas, and exhibit normal reproductive patterns, longevity and behavior [49]. We determined the behavioral effect of HSPB1 over-expression on Npc1 deficiency by measuring the time to traverse a balance beam. Purkinje cell specific null mutants (*Npc1 flox/-;Pcp2-Cre*), but not littermate controls (*Npc1 flox/+;Pcp2-Cre*), displayed a progressive, age-dependent behavioral impairment beginning at 10 weeks (Fig 4A), consistent with our previous study [33]. HSPB1 over-expression significantly rescued motor performance in mice at 10 and 15 weeks of age (Fig 4A). Previous work has demonstrated that this motor task is a sensitive measure of Purkinje cell loss in Npc1 deficient mice [33]. To determine the extent to which HSPB1 over-expression improved neuron survival, we examined the density of Purkinje cells in the cerebellar midline of mice at 11 weeks. This analysis revealed that HSPB1 over-expression significantly rescued Purkinje cell density in posterior (lobules VIII-X) but not anterior cerebellar lobules (Fig 4B and 4C–4J). Purkinje cell rescue in posterior lobules was confirmed by immunofluorescence staining for calbindin, a marker of Purkinje cells (Fig 4G versus 4I). This rescue was associated with the expression of HA-tagged HSPB1 transgene (Fig 4H versus 4J). Transgene expression was also noted in anterior lobules, suggesting that HSPB1 over-expression alone was insufficient to account for effects on neuron survival. The HSPB1 transgene did not alter the accumulation of ubiquitinated proteins or filipin-positive unesterified cholesterol in Purkinje cells of posterior lobules (S2 Fig). We conclude that exogenous HSPB1 protects Purkinje cells in posterior lobules and delays the onset of behavioral impairment, without altering the aberrant accumulation of proteins or cholesterol.

**Fig 5:**
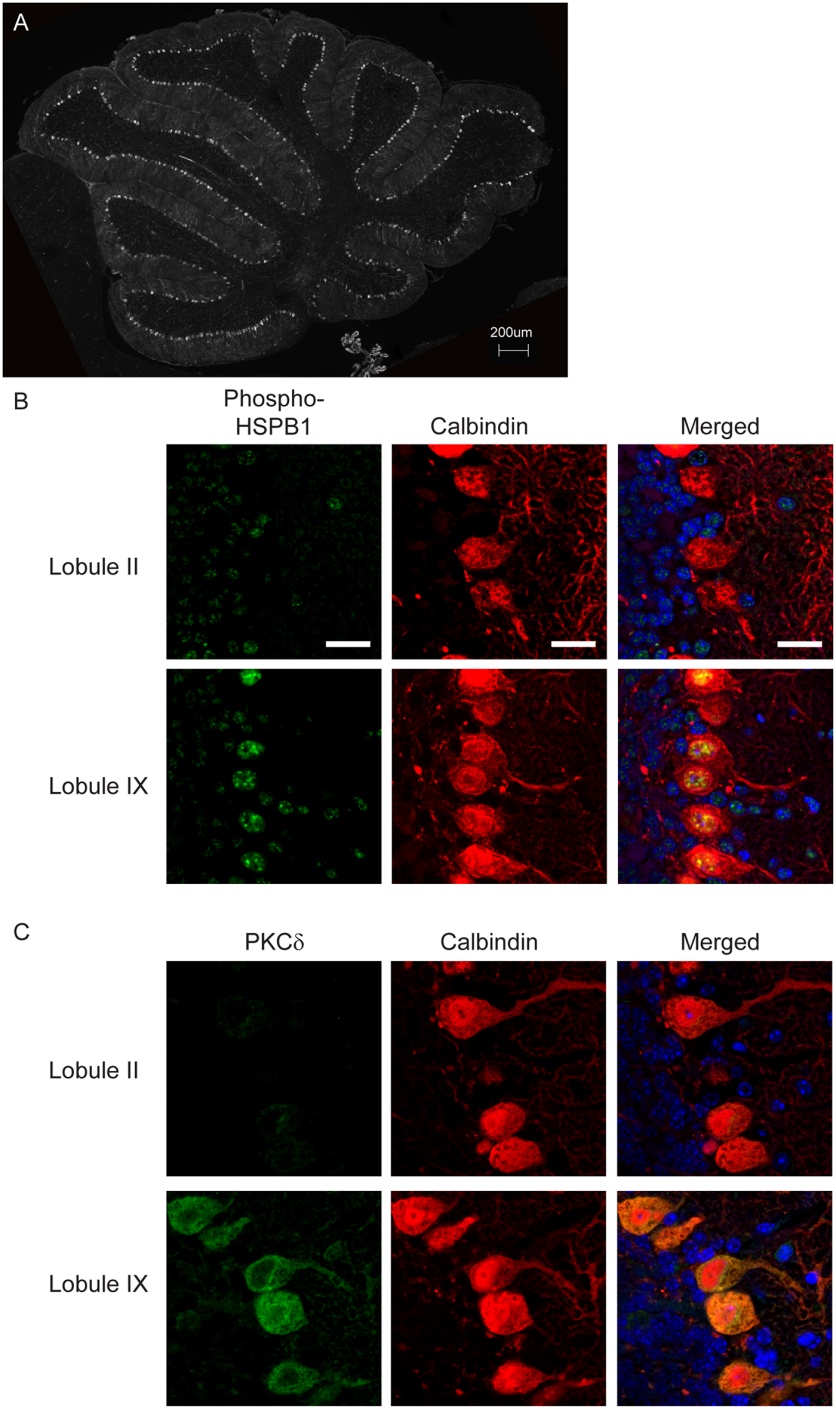
PKCδ and phosphorylated HSPB1 are co-expressed in Purkinje cells in posterior lobules. **(A)** Transgenic HSPB1 (HA) in the cerebellar midline of 7-week-old *Npc1 flox/–*, *Pcp2-Cre*, *HSPB1* mice. Scale bar = 200 μm. **(B, C)** Expression of phospho-HSPB1 (serine 15, *in green*, *panel B*) and PKCδ (*in green*, *panel C*) were examined in Purkinje cells (calbindin, *in red*) in the cerebellar midline of *Npc1 flox/–*, *Pcp2-Cre*, *HSPB1* mice at 7 weeks of age. Nuclei were stained by DAPI. *Top row*, lobule II; *bottom row*, lobule IX. Scale bar = 20 μm.

To further explore the basis of the beneficial effects of HSPB1 on select Purkinje cell subpopulations, we first evaluated whether the transgene was uniformly expressed. HA staining of the cerebellar midline confirmed diffuse reactivity of Purkinje cells in 7 week old *Npc1 flox/-;Pcp2-Cre*, *HSPB1* mice (Fig 5A). We next considered the possibility that HSPB1 was differentially activated in cerebellar lobules. Because phosphorylation of HSPB1 influences its ability to promote neuronal survival in vitro (Fig 3E), we examined HSPB1 phosphorylation state in Purkinje cells using phospho-HSPB1 [pS15] immunofluorescence. Strikingly, only Purkinje cells in posterior lobules were positive for phospho-HSPB1 (Fig 5B) despite the fact that the transgene was diffusely expressed (Fig 5A). Intriguingly, our expression analysis identified restricted expression of the HSPB1 kinase PKCδ [50–52] to Purkinje cells in the posterior lobules (Fig 2A, Table 1), a finding that was confirmed by immunofluorescence staining (Fig 5C). Taken together, these data indicated that phosphorylation of HSPB1 was tightly associated with Purkinje cell rescue in animals expressing the transgene.

**Fig 6:**
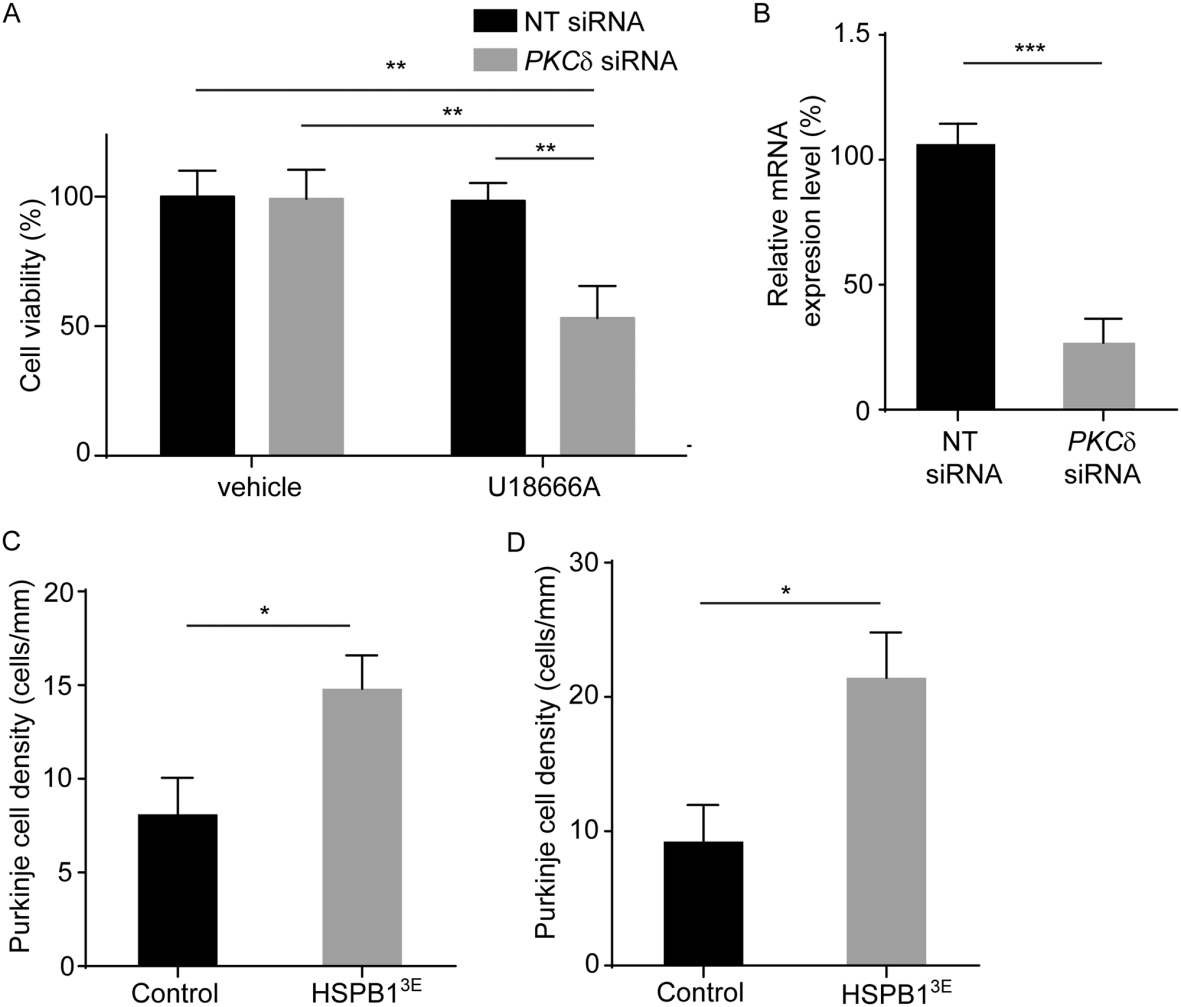
PKCδ and HSPB1 phosphorylation increase cell viability. **(A)** HeLa cells were transfected with non-targeted (NT) or *PKCδ* siRNA and then treated with 3 μg/ml U18666A or vehicle for 24 hrs. XTT assay was performed to measure cell viability. Data are mean ± SD, **p<0.01. **(B)** *PKCδ* expression was determined by quantitative real-time PCR. Data are mean ± SD, ***p<0.001. **(C, D)** 6-week-old *Npc1 flox/-*, *Pcp2-Cre* mice were injected with AAV2 expressing HSPB1-3E or control vector and then examined at 10 weeks of age. Quantification of Purkinje cell density in lobules VI **(C)** and VII **(D)** of midline cerebellar sections. Data are mean ± SEM, *n* = 4 mice/group. *p<0.05.

We sought to additionally explore the functional importance of HSPB1 phosphorylation in mediating cell survival in models of NPC. Prior studies have shown that PKCδ phosphorylates HSBP1 at Ser-15 and Ser-86 to reduce apoptosis [50–52], suggesting that these two proteins may act together to promote cell survival. To determine whether this pathway was active in cellular models of NPC, we knocked down the expression of PKCδ with targeted siRNA and then treated cells with U18666A. We found that diminished PKCδ expression significantly increased the sensitivity of cells to U18666A-mediated toxicity (Fig 6A and 6B), similar to the effect of *HSPB1* gene knockdown (Fig 3B and 3C). To evaluate *in vivo* activity of the phosphorylated form of HSBP1, we used an adeno-associated virus serotype 2 (AAV2) vector to over-express phosphomimetic HSPB1-3E. Transgene and control viral vectors were injected into the deep cerebellar nuclei of *Npc1 flox/-;Pcp2-Cre* mice at 6 weeks and animals were examined four weeks post-infection. Gene delivery as visualized with the 6x-myc tag was strong and consistent in the central and posterior lobules of the cerebellar midline. Quantification of Purkinje cell density confirmed a significant rescue in the central lobules VI and VII, as well as in the posterior lobule VIII, of mice expressing HSPB1-3E compared to controls (Fig 6C and 6D and S3 Fig). As Purkinje cell survival was not significantly rescued in these central lobules by transgenic expression of wild type HSPB1 (Fig 4B), we conclude that the phosphorylated form of HSPB1 was active in promoting Purkinje cell survival in the NPC cerebellum.

### Hspb1 knockdown exacerbates Purkinje cell loss

**Fig 7:**
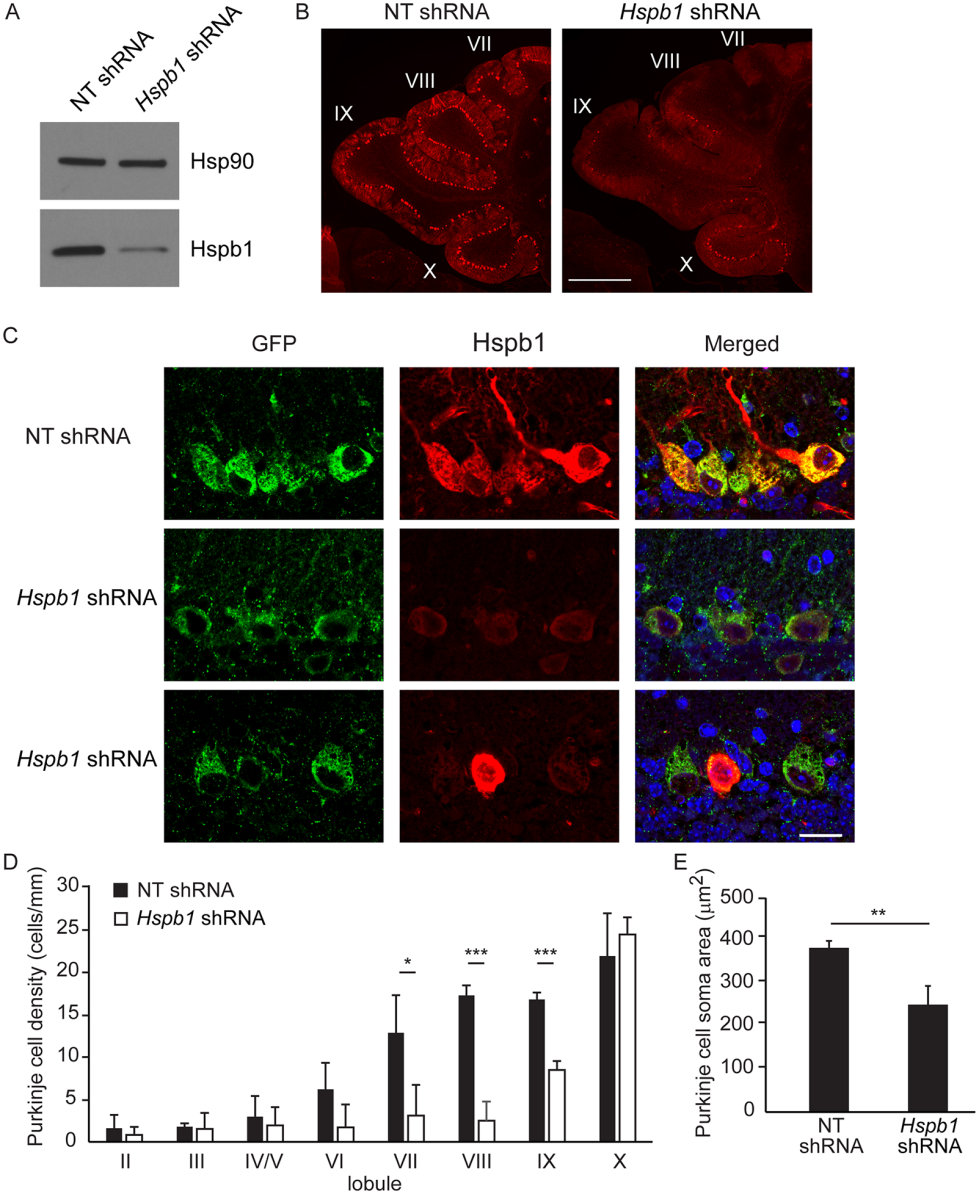
*Hspb1* knockdown exacerbates Purkinje cell loss. **(A)** NIH3T3 cells were transfected with non-targeted (NT) or *Hspb1* shRNA. After 24 hrs, cells were heat shocked (43°C for 90 min) and protein lysates were collected after 24 hrs. Hspb1 expression was determined by western blot. Hsp90 controls for loading. **(B–D)** 7-week-old *Npc1 flox/-*, *Pcp2-Cre* mice were injected with AAV2 expressing non-targeted shRNA (NT shRNA) or *Hspb1* shRNA, and then examined at 13 weeks of age. **(B)** Immunofluorescent staining of Purkinje cells (calbindin) in the cerebellar midline. *Left panel*, NT shRNA; *right panel*, *Hspb1* shRNA. Roman numerals identify lobules. Scale bar = 500 μm. **(C)** Expression of AAV2-encoded GFP (green) and endogenous Hspb1 (red). *Top row*, NT shRNA; *middle and bottom rows*, *Hspb1* shRNA. Nuclei were stained by DAPI. Scale bar = 20 μm. **(D)** Quantification of Purkinje cell density by lobule. Data are mean ± SD, *n* = 3 mice/genotype. *p<0.05, ***p<0.001. **(E)** Quantification of Purkinje cell soma area in lobule X. Data are mean ± SD, *n* ≥ 91 cells/genotype. **p<0.01.

Our over-expression studies demonstrated that HSPB1 delays motor impairment and Purkinje cell loss in posterior cerebellar lobules. We next sought to determine the effects of Hspb1 knockdown in the NPC mouse cerebellum. The feasibility of this approach was supported by prior work demonstrating that *Hspb1* null mice are viable and fertile, without obvious morphological abnormalities [53]. To accomplish gene knockdown, we used an AAV2 vector to produce a short hairpin RNA (shRNA) driven by the U6 promoter. *Hspb1* shRNA was cloned into an AAV2 shuttle plasmid (pFBAAV/mU6mcsCMVeGFP). To initially confirm knockdown efficiency, NIH3T3 cells were transfected to express non-targeted (NT) or *Hspb1* shRNA, heat shocked, and analyzed by western blot (Fig 7A). These targeted and control shRNA clones were then used for virus generation, and injected into the deep cerebellar nuclei of *Npc1 flox/-;Pcp2-Cre* mice at 7 weeks. Animals were examined six weeks post-infection. At this time point, calbindin staining for Purkinje cells was markedly diminished in the posterior cerebellar lobules of mice receiving *Hspb1* shRNA (Fig 7B). We confirmed viral transduction of remaining Purkinje neurons by GFP staining and assessed knockdown efficiency by Hspb1 staining. We observed diffuse GFP reactivity of Purkinje cells in mice expressing NT and *Hspb1* shRNA, whereas Hspb1 staining was specifically diminished by *Hspb1* shRNA (Fig 7C). Quantification of Purkinje cell density confirmed a significant exacerbation of neuron loss in central and posterior cerebellar lobules (lobules VII-IX) of mice expressing *Hspb1* shRNA (Fig 7D). Furthermore, although Purkinje cell density was not altered in lobule X, Hspb1 knockdown significantly diminished soma size (Fig 7E). These data indicate that Hspb1 knockdown exacerbates Purkinje cell degeneration due to NPC1 deficiency.

## Discussion

Many progressive neurological diseases are characterized by the selective vulnerability of neuronal populations, yet mechanisms underlying this phenomenon remain poorly characterized. Here, we sought to identify potential modifier genes that influence the susceptibility of neurons to disease. Using NPC disease as a model for the study of selective neuronal vulnerability, we demonstrate that one of the candidate genes we identified, *HSPB1*, promotes neuronal survival in cellular model systems through a mechanism that likely involves phosphorylation-dependent inhibition of apoptosis. Additionally, we show that HSPB1 over-expression *in vivo* slows the progression of motor impairment and diminishes cerebellar Purkinje cell loss. The neuroprotection from Npc1 deficiency afforded by HSPB1 over-expression in mice was associated with HSPB1 phosphorylation and expression of the kinase PKCδ. We confirmed the modulatory effect of Hspb1 on Purkinje cell degeneration *in vivo*, as knockdown by *Hspb1* shRNA significantly enhanced neuron loss. This effect of *Hspb1* gene knockdown was particularly robust, resulting in Purkinje cell degeneration in posterior lobules (VII-IX) that approached the severity observed in anterior cerebellar lobules. Although diminished Hspb1 expression did not trigger Purkinje neuron loss in lobule X, we observed a significant decrease in soma size, a compensatory change reported in other degenerative ataxias that influences membrane excitability [54]. These results highlight the use of bioinformatics tools to uncover pathways leading to neuronal protection in neurodegenerative disorders.

HSPB1 is a multifunctional protein with documented roles in actin stability, protein folding, oxidative damage, and apoptosis [40]. Interestingly, HSPB1 is a direct inhibitor of apoptosis at multiple levels, through binding and sequestration of cytochrome c [55] and caspase-3 [56], and inhibition of Bax activation [57] and DAXX signaling [58]. The phosphorylation state required for most of these activities is unknown, with the exception of DAXX inhibition, which requires phosphorylated HSPB1 [58]. Recently, phosphomimetic mutants of HSPB1 were shown to protect against a broad array of apoptosis-inducing stimuli, while non-phosphorylatable mutants showed no protection against some stimuli and only mild protection against others, suggesting that anti-apoptotic activities of HSPB1 are primarily attributable to the phosphorylated species [59]. Although both phosphorylated and dephosphorylated HSPB1 have chaperone activity [60, 61] and prevent oxidative damage [62], it is less likely that these functions play a primary role in exerting beneficial effects in NPC models. This conclusion is based on the tight association that we observed between HSPB1 phosphorylation and neuroprotection, and the finding that neuronal rescue is not associated with diminished accumulation of ubiquitinated proteins. Instead, we favor a model in which HSPB1 acts through an anti-apoptotic mechanism. Our findings suggest that strategies that promote HSPB1 expression or phosphorylation may diminish the rate of cerebellar degeneration in NPC disease.

Interestingly, we also identified the expression of PKCδ in disease-resistant Purkinje cells. While this kinase has been shown to phosphorylate Hspb1 at Ser-15 and Ser-86 to reduce apoptosis [50–52], it is possible that other kinases also contribute to the regulation of Hspb1 activity in the cerebellum. To initially explore this possibility, we examined the expression of kinases that have been previously reported to phosphorylate HSPB1. This includes protein kinase D (PKD) [63], mitogen-activated protein kinase-activated protein kinase-2 and 3 (MAPKAPK2/3) [64, 65] and p38 mitogen-activated protein kinase (p38 MAPK) [66, 67]. According to information in the Allen Brain Atlas, PKD1, PKD2, and PKD3 and MAPKAPK3 are not expressed in the cerebellum, while MAPKAPK2 and p38 MAPK are expressed by all Purkinje neurons. The association between restricted expression of PKCδ, the occurrence Hspb1 phosphorylation, and the pattern of Purkinje neuron survival prompts us to favor PKCδ as an important regulator of the survival benefits mediated by Hspb1. Moreover, we note that the gene encoding a phosphatidylinositol-specific phospholipase C, *Plcxd2*, is also expressed by resistant Purkinje neurons in the posterior cerebellar lobules (Table 1). This observation raises the possibility that both the regulatory components and effectors of this pro-survival pathway are preferentially expressed by the subset of disease-resistant Purkinje neurons.

Our identification of candidate disease modifying genes relied on *in situ* hybridization data available in the Allen Brain Atlas. Published studies have similarly mined data from this public database to uncover biologically important gene expression variation [68, 69]. For guidance in our study, we looked to tools developed for the analysis of microarray data, where studies of differential gene expression are commonplace. Several caveats exist when applying our strategy to Allen Brain Atlas data. First, this method is heavily dependent upon manual curation as standard statistical tests yielded high false positive rates. These were variably due to signals generated by other cell types that fell within or adjacent to the region of interest, or artifacts and noise on the *in situ* hybridization images. Second, while the majority of differentially expressed genes were identified by both *t*-test and SAM, others were found only by one method. Therefore, it was necessary to combine the use of both approaches, and it remains possible that some differentially expressed genes were not discovered by either. To streamline future studies, a more robust method for working with Allen Brain Atlas data may need to be developed. Despite these technical limitations, our study provides proof of concept for the use of Allen Brain Atlas data to identify therapeutic targets in neurodegenerative diseases.

## Materials and Methods

### Ethics statement

All animal procedures were approved by the University of Michigan Committee on the Use and Care of Animals (protocol number PRO00006114). Euthanasia of mice was performed by anesthesia overdose followed by induction of bilateral pneumothorax or removal of vital organs.

### Antibodies

Antibodies used in this study were anti-GAPDH (Santa Cruz, sc-25578), anti-calbindin (Sigma-Aldrich, c2724), anti-HSPB1 (abcam, ab5579), anti-Hspb1 (Enzo Life Sciences, SPA-801), anti-HSPB1 phospho-Ser 15 (Novus, NBP1-60864), anti-PKCδ (Fisher, BDB610397), anti-hemagglutinin (HA) (Covance, 16B12), anti-GFP (Novus, NB 100–1770), anti-Hsp90 (Santa Cruz, sc-7947) and anti-ubiquitin (Dako, Z0458).

### Mice

Mice containing the *Npc1* floxed (exon 9) [33] and null alleles [70], and transgenic mice expressing the *Cre* transgene driven by the *Pcp2* promoter [71] were generated and genotyped as described previously. Transgenic mice over-expressing hemagglutinin tagged human HSPB1 were from Dr. Jacqueline de Belleroche (Imperial College, London, UK) [49]. All lines were backcrossed to C57BL6/J for ≥10 generations. All animal procedures were approved by the University of Michigan Committee on the Use and Care of Animals.

### Cell culture

All cell lines were cultured at 37°C with 5% CO_2_. HeLa cells were maintained in DMEM (Gibco, 11965–092) supplemented with 10% FBS, 1X penicillin, streptomycin, and glutamine (Gibco, 10378–016). Human skin fibroblasts GM03123 from an NPC patient and GM08399 from an age and sex matched control (Coriell Cell Repositories) were maintained in MEM (Gibco, 10370–021) supplemented with 15% FBS, 1X penicillin, streptomycin, and glutamine (Gibco). To manipulate HSPB1 expression, cells were transfected with ON-TARGET*plus* SMART pool human HSPB1 or non-targeting control (Dharmacon). HeLa cells were transfected using the DharmaFECT reagent (Dharmacon), according to the manufacturer’s instructions. Fibroblasts were transfected by electroporation with the Lonza Nucleofector normal human dermal fibroblast kit. To reduce PKCδ expression, HeLa cells were transfected with ON-TARGET plus SMART pool PKCδ siRNA (Dharmacon, L-003524-00-0005) or ON-TARGET plus non-targeting pool (Dharmacon, D-001810-10-05), using TransIT-X2 (Mirus).

### Genome-wide expression profiling

The Expression Energy Volume for each gene in the Allen Mouse Brain Atlas was downloaded via the Allen Brain Atlas API [34]. These data were then reorganized into a single expression matrix and filtered to include locations corresponding to the regions of interest, cerebellar lobules X and II/III, and extending laterally 1400 microns from the midline. This data matrix was then loaded into TM4 MultiExperiment Viewer software [72], in which differential expression between regions of interest was determined by Student’s *t*-test and Significance Analysis of Microarrays (SAM) [35]. The top 1000 genes returned by each method were manually verified by direct inspection of *in situ* hybridization data on the Allen Brain Atlas website in midline and several adjacent sagittal sections. Criteria for validation were (1) expression present in the Purkinje cell layer in at least one region of interest, and (2) differential expression between regions of interest.

### Western blotting

Cells were lysed in RIPA buffer (Thermo Scientific) containing Complete protease inhibitor (Roche) and Halt phosphatase inhibitor (Thermo Scientific). Samples were electrophoresed through a 10% SDS-PAGE gel, and then transferred to nitrocellulose membranes (BioRad) using a semidry transfer apparatus. Primary antibodies were anti-HSPB1 (1:1000), anti-Hsp90 (add dilution) and anti-GAPDH (1:5000). HRP-conjugated secondary antibodies were from BioRad. Blots were developed using ECL (Thermo Scientific) or TMA-6 (Lumigen) chemilluminescent reagents, following manufacturers’ protocols.

### Gene expression analysis

Total RNA was isolated from HeLa cells using TRIzol (Invitrogen). cDNA was synthesized using the High Capacity cDNA Archive Kit (Applied Biosystems). Quantitative real-time PCR was performed on 100 ng of cDNA in triplicate, using primers and probes for PKCδ (cat # 4453320) and 18S rRNA (Applied Biosystems). Threshold cycle (Ct) values were determined using an ABI Prism 7900HT Sequence Detection System. Relative expression values were normalized to 18S rRNA.

### Apoptosis and viability assays

Caspase-3 activity in HeLa cells was determined by assaying DEVDase activity in cell lysates using the ApoTarget caspase 3 / CPP32 fluorimetric protease assay kit (Biosource) according to the manufacturer’s instructions. Fluorescence was measured using a SpectraMax Gemini EM plate reader (Molecular Devices). NPC fibroblasts were stained with Hoechst (Immunocytochemistry Technologies). Cells were counted in five randomly selected fields per transfection at 200x magnification and scored for chromatin condensation. The viability of primary mouse cortical neurons and HeLa cells was determined by XTT assay (Cell Proliferation Kit II, Roche). XTT reagent and activation reagent were mixed at a ratio of 50:1 and added to cultures. After incubating for 4 hrs at 37°C, absorbance at 490 nm and 650 nm was measured using a SpectraMax Gemini EM plate reader (Molecular Devices).

### Primary cortical neuron culture

Cortices from P0 C57BL6/J mouse pups were dissected free of meninges, minced, and then dissociated and cultured as described previously [73]. Neurons were plated in poly-D-lysine (Millipore) treated 96-well plates at a density of 6x10^4^ cells per well. Cytosine arabinoside (Sigma) was added to the culture media the following day at a final concentration of 5 M to prevent glial growth. U18666A was added at 2.5 μg/ml at 7 div to induce lipid storage.

### Viral vectors

A lentiviral expression clone of human *HSPB1* with a C-terminal FLAG tag was obtained from Genecopoeia. Serine-to-alanine and serine-to-glutamate mutations were introduced at serines 15, 78, and 82 using the QuikChange Lightning Multi Site-Directed Mutagenesis kit (Stratagene). Wild type *HSPB1*, *HSPB1-*3A, *HSPB1-*3E, and empty vector plasmids were packaged into feline immunodeficiency virus (FIV) vectors by the Iowa Vector Core. Viral infection of cultured primary neurons was performed at 10 MOI, followed by a 75% media change four hours after infection. For in vivo gene over-expression, *HSPB1-*3E with a 6x-myc tag was cloned into pFBAAV/CMVmcspA. For gene knock-down, Hspb1 shRNA was designed and cloned by the Iowa Vector Core. The target region in the Hspb1 sequence was analyzed using siSPOTR and potential miRNA target sequences of 21 nucleotides were identified based on low GC content and other factors, as described [74]. Five potential target sequences were cloned in pFBAAV/mU6mcsCMVeGFP. Knockdown efficiency was tested in NIH3T3 cells. The most efficient plasmid was used in producing AAV2/1mU6miHspb1-CMVeGFP or AAV2/1CMVHSPB1-3E triple transfection virus. Non-targeted virus, AAV2/1mU6-miSafe-CMV eGFP, was used as a control. Before injection, virus was dialyzed at 4°C for 3hrs against 7,000 MWCO Slide -A-Lyzer mini-dialysis units (Thermo Scientific) in a custom buffer formulation distributed through the Gene Transfer Vector Core in University of Iowa.

### Stereotaxic cerebellar viral delivery

Stereotaxic administration of AAV2 was performed on 7 week-old *Npc1 flox/-*, *Pcp2-Cre* mice placed under anesthesia using a mixture of O_2_ and isoflurane (dosage 4% for induction, 1.5% maintenance). Mice received bilateral intracerebellar injections (either one or two sites/hemisphere) of virus. For each injection, ~1.4 x 10^12^ vg/ml of virus (4 μl) was delivered to the medial or lateral cerebellar nucleus at an infusion rate of 0.5 μl/min using a 10-μl Hamilton syringe (BD). One min after the infusion was completed, the micropipette was retracted 0.3 mm and allowed to remain in place for 4 min prior to complete removal from the mouse brain. When two injections sites per hemisphere were used, anterior–posterior coordinates were calculated separately for medial and lateral injection into each cerebellar hemisphere. The coordinates for the medial injection were -6.4 mm anterior-posterior, ±1.3 mm medial-lateral and 1.9 mm dorsal-ventral as measured from bregma. The coordinates for the lateral injection were -6.0 mm anterior-posterior, ±2.0 mm medial-lateral and 2.2 mm dorsal-ventral as measured from bregma. When a single injection per hemisphere was used, the coordinates for the injection were -6.2 mm anterior-posterior, ±0.9 mm medial-lateral and 2.2 mm dorsal-ventral as measured from bregma.

### Immunofluorescence staining

5 μm sections from brains embedded in paraffin were deparaffinized with xylenes and ethanol. Sections were boiled in 10 mM sodium citrate, pH 6, for 10 min for antigen retrieval. After washing with water, sections were blocked with 5% goat serum and 1% BSA in PBS for 1 hr and then incubated in primary antibody (calbindin 1:500, PKCδ 1:50, Hspb1 1:100, HA 1:200, phospho-Hspb1 1:50, GFP 1:100, ubiquitin 1:200) diluted in 1.5% blocking solution overnight at 4°C. Sections were subsequently incubated in secondary antibodies conjugated to Alexa Fluor 594 or 488 for 2 hrs and mounted with mounting medium including DAPI (Vector Lab, H-1200). Images were captured on an Olympus FluoView 500 Confocal microscope.

### Behavioral testing

Motor function was measured by balance beam test. Mice at 4 weeks of age were trained on three consecutive days to cross a 6 mm wide beam suspended at 50 cm. Mice were then tested in triplicate at 5, 10, 15 and 20 weeks of age. Data are reported as average time to traverse the beam, allowing a maximum of 25 sec and scoring falls as 25 sec.

### Morphological analysis

Purkinje cell density was quantified in midline sagittal sections stained with hematoxylin and eosin or calbindin staining. Purkinje cells were recognized as large cells with amphophilic cytoplasm, large nuclei with open chromatin and prominent nucleoli that were located between the molecular and granular layers or as calbindin positive cells. The number of cells was normalized to the length of the Purkinje layer, as measured by NIH ImageJ software. For analysis of Purkinje cell soma size, calbindin staining was used to define the cell soma. The cell soma was selected and measured by NIH ImageJ, and pixel size was converted to μm^2^ using the scale bar as a calibration standard.

### Filipin staining

Mouse brain tissue embedded in Optimal Cutting Temperature (OCT) compound (Tissue-Tek) was sectioned at 10 μm in midline. The sections were rinsed with PBS, and fixed with 4% paraformaldehyde for 30 min. After washing with PBS, the sections were incubated with 1.5 mg/ml glycine for 10 min, washed with PBS and stained with 0.05 mg/ml filipin and 10% FBS in PBS for 2 hrs at room temperature. Filipin images were captured with the UV filter set on an Olympus FluoView 500 confocal microscope. Representative images are from one of three mice per genotype.

### Statistics

Statistical significance was assessed by unpaired Student’s *t* test (for comparison of two means) or ANOVA (for comparison of more than two means). The Newman-Keuls post hoc test was performed to carry out pairwise comparisons of group means if ANOVA rejected the null hypothesis. Statistical analyses were performed using the software package Prism 6.02 (GraphPad Software). *P* values less than 0.05 were considered significant. Statistical analysis of gene expression data was performed using TM4 MultiExperiment Viewer software [72]. For these calculations, statistical significance was determined using Student’s *t*-test with Bonferroni correction for multiple comparisons and Significance Analysis of Microarrays (SAM) [35].

## Supporting Information

S1 Fig Genes selectively expressed in posterior lobules of the cerebellar midline. *In situ* hybridization images from the Allen Brain Atlas.
(TIF)

S2 Fig HSPB1 over-expression does not rescue accumulation of ubiquitinated proteins or unesterified cholesterol. Sections of the cerebellar midline were examined from mice at 11 weeks of age. **(A)** Immunofluorescent staining for calbindin (green) and ubiquitin (red); nuclei were stained by DAPI. **(B)** Immunofluorescent staining for calbindin (red) and filipin (blue). Scale bar = 20 μm.
(TIF)

S3 Fig HSPB1-3E over-expression rescues Purkinje cell loss in lobule VIII. 7-week-old *Npc1 flox/-*, *Pcp2-Cre* mice were injected with AAV2 expressing HSPB1-3E or control vector and then examined at 13 weeks of age. Quantification of Purkinje cell density **(A)** and soma size **(B)** in lobule VIII of midline cerebellar sections. Data are mean ± SD, *n* = 3 mice/group. *p<0.05.
(TIF)
