# Supplementary figures and images for: Heat Shock Protein Beta-1 Modifies Anterior to Posterior Purkinje Cell Vulnerability in a Mouse Model of Niemann-Pick Type C Disease
Source: PLoS Genet. 2016 May 6;12(5):e1006042. doi: 10.1371/journal.pgen.1006042 (PMC4859571; doi:10.1371/journal.pgen.1006042)

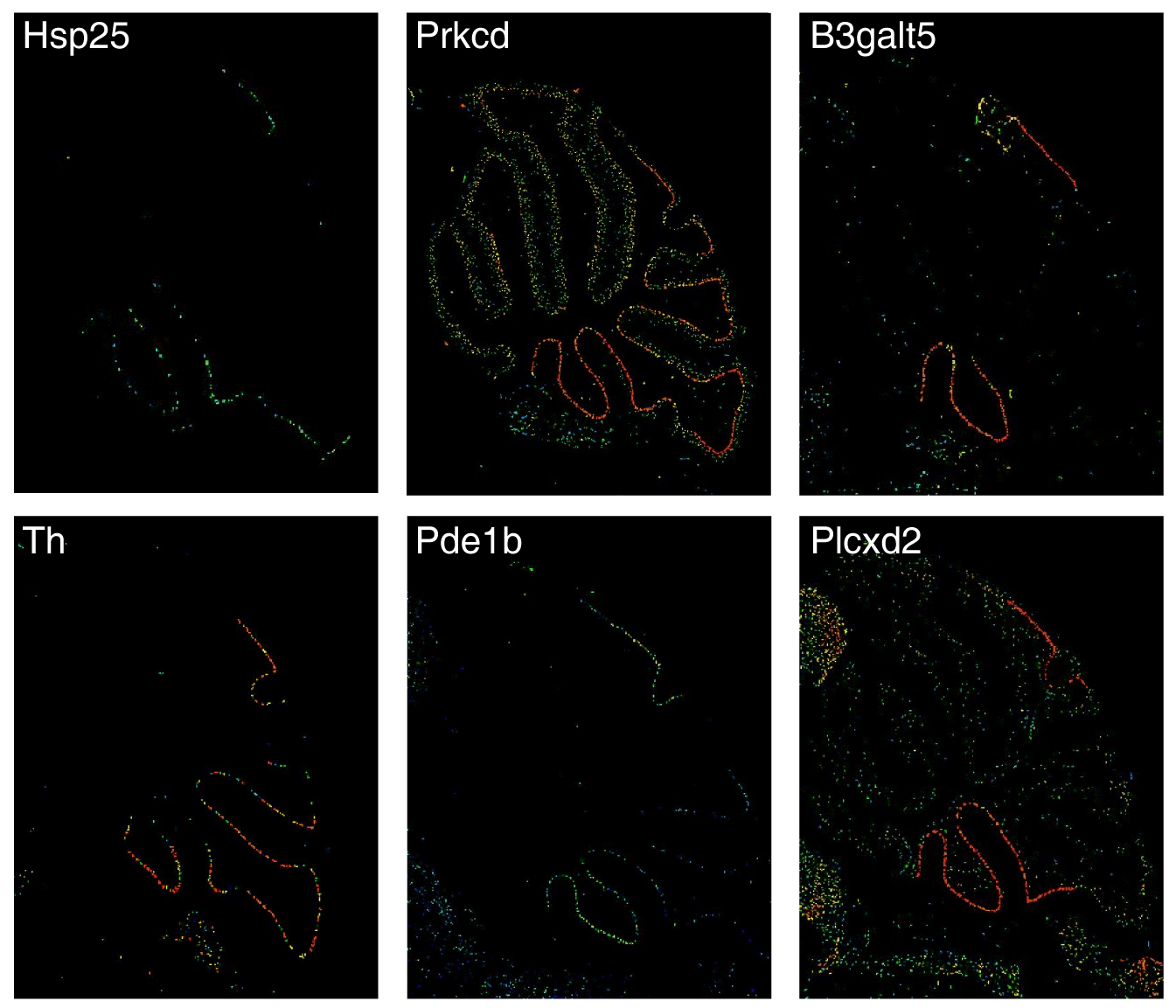

Supplement: S1 Fig — In situ hybridization images from the Allen Brain Atlas. (TIF) [file pgen.1006042.s001.tif]

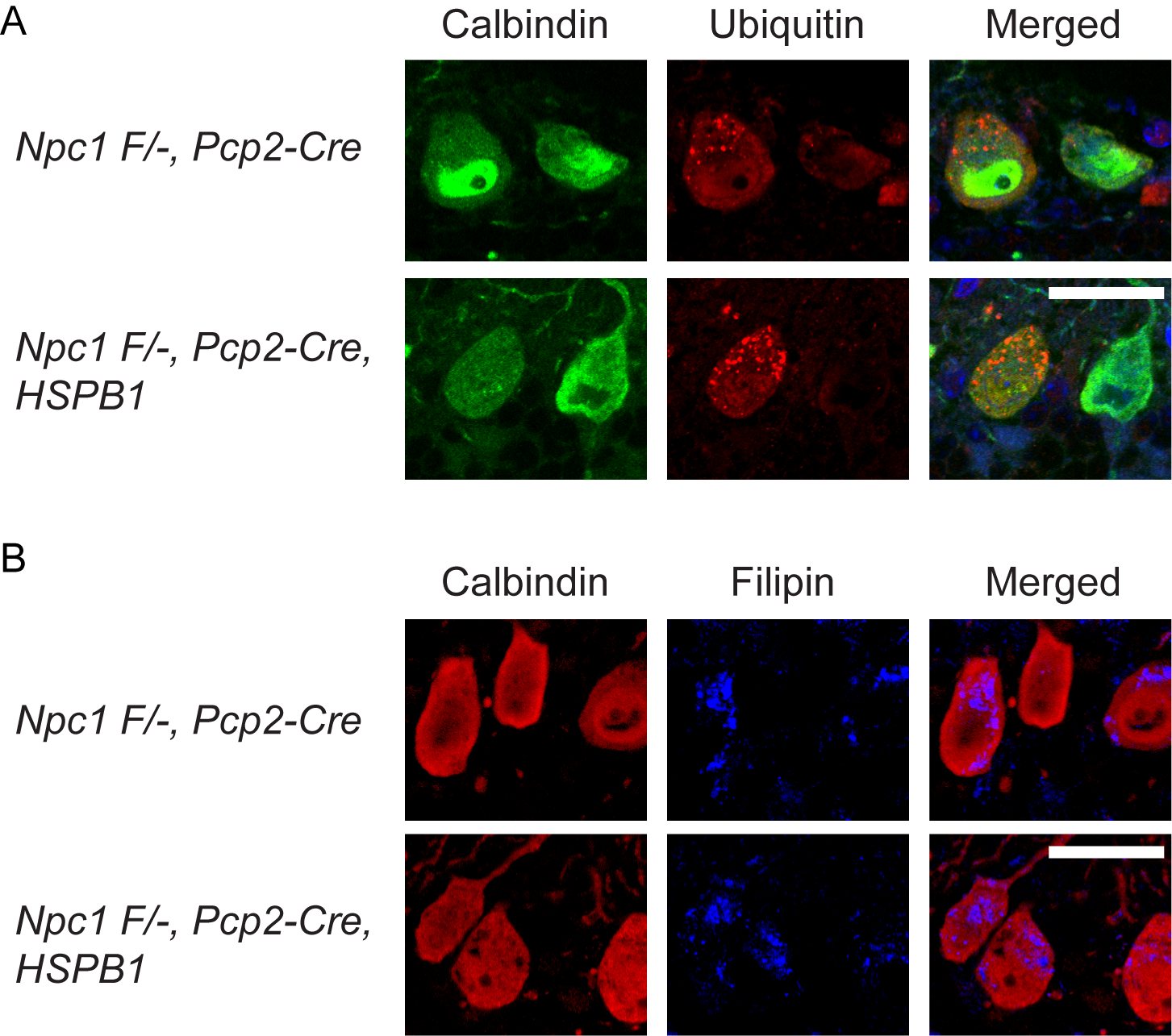

Supplement: S2 Fig — Sections of the cerebellar midline were examined from mice at 11 weeks of age. (A) Immunofluorescent staining for calbindin (green) and ubiquitin (red); nuclei were stained by DAPI. (B) Immunofluorescent staining for calbindin (red) and filipin (blue). Scale bar = 20 μm. (TIF) [file pgen.1006042.s002.tif]

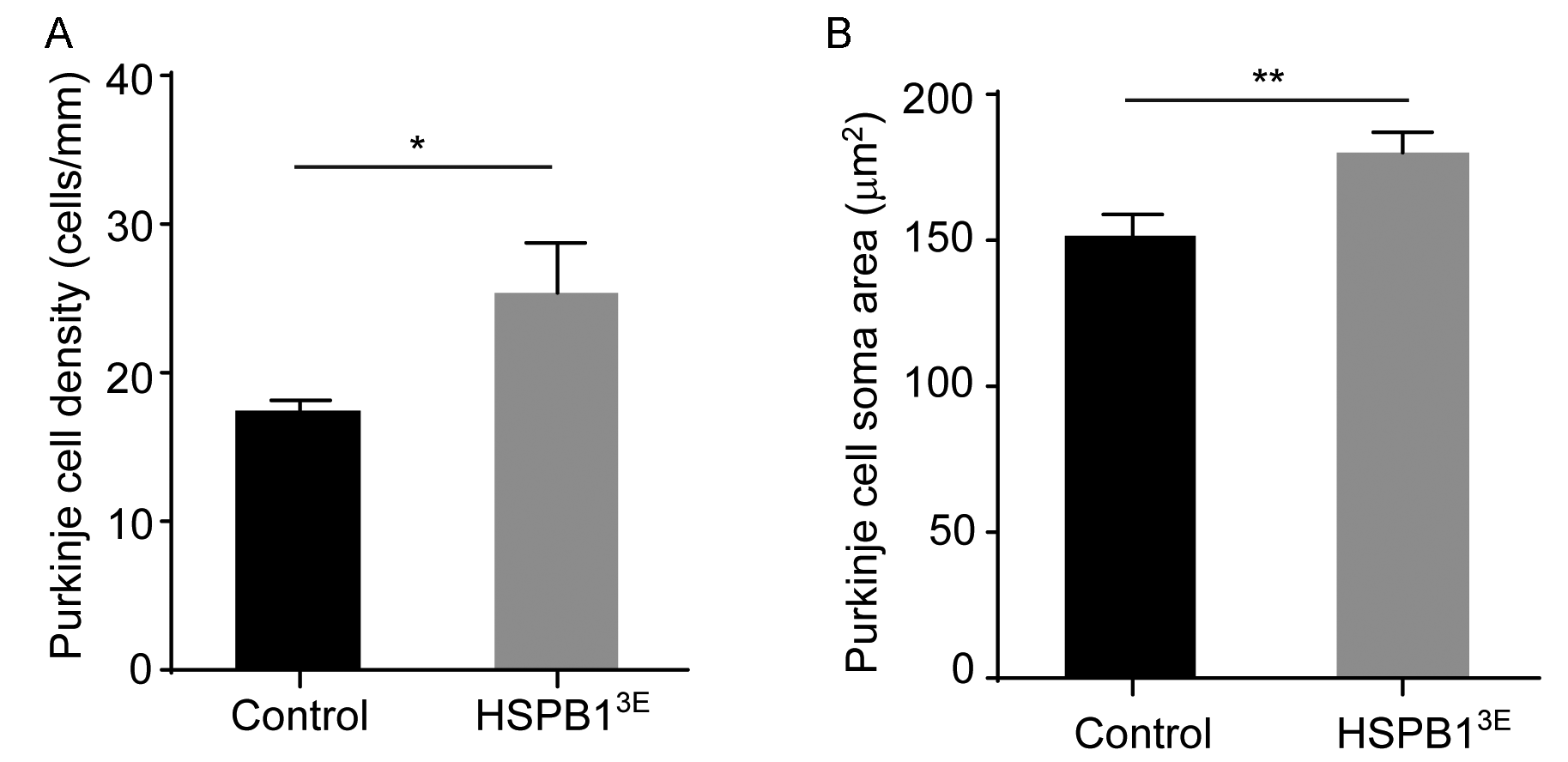

Supplement: S3 Fig — 7-week-old Npc1 flox/-, Pcp2-Cre mice were injected with AAV2 expressing HSPB1-3E or control vector and then examined at 13 weeks of age. Quantification of Purkinje cell density (A) and soma size (B) in lobule VIII of midline cerebellar sections. Data are mean ± SD, n = 3 mice/group. *p<0.05. (TIF) [file pgen.1006042.s003.tif]
